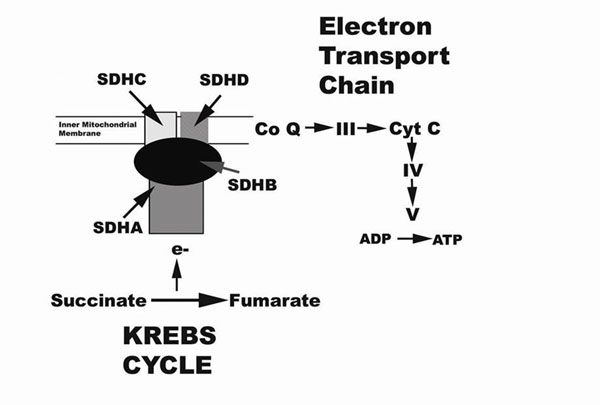# Use of SDHB immunohistochemistry to identify germline mutations of SDH genes

**DOI:** 10.1186/1897-4287-10-S2-A7

**Published:** 2012-04-12

**Authors:** AJ Gill

**Affiliations:** 1 Royal North Shore Hospital, Australia

## 

Pheochromocytomas and paragangliomas occur sporadically but are commonly associated with the von Hippel Lindau (VHL) syndrome, multiple endocrine neoplasia type 2 (MEN2), neurofibromatosis type 1 (NF1) and germline mutations of succinate dehydrogenase B (SDHB), C (SDHC) or D (SDHD). It is therefore recommended that genetic testing be considered if not performed in all cases of even apparently sporadic pheochromocytomas or paragangliomas. Recently it has been demonstrated that immunohistochemistry (IHC) for SDHB is negative in all SDH mutated paragangliomas regardless of whether the B,C or D subunit is involved [1,2]. Furthermore some clearly syndromic paragangliomas without known genetic mutation (including but not limited to those which occur in the Carney Triad) are identified by negative staining for SDHB [3].

Although historically the renal tumours occurring in the setting of SDHB mutation were usually classified as conventional clear cell carcinoma or oncocytoma, they actually display a unique morphology (unrecognized until know) and which can be confirmed by immunohistochemistry.

The GISTs occurring in SDH mutation and Carney Triad are also unique and demonstrate quite a different morphology, natural history and molecular pathogenesis compared to other GISTs occurring in adults (but similar to most GISTs occurring in childhood). We call this unique subtype of GIST the type 2 GIST. Briefly type 2 GISTs arise in the stomach, show an epithelioid morphology, are often multifocal, commonly show lymph node metastasis, are wild type for KIT and PDGFR, have a prognosis not predicted by size and mitotic rate, never respond to imatinib but demonstrate an indolent growth despite the presence of frequent metastases [3,5].

We recommend that all paragangliomas, GISTs which potentially display type 2 morphological or clinical features and renal carcinomas which display the unique morphology we described should undergo immunohistochemistry for SDHB. Negative staining for SDHB indicates an abnormality of the mitochondrial complex 2 and is an absolute indication for formal genetic testing. We perform and interpret SDHB immunohistochemistry of archived formalin fixed paraffin embedded tissue in a manner analogous to MSI testing in colon cancer. In the setting of paraganglioma or renal carcinoma negative staining almost always indicates germline SDHB,SDHC or SDHD mutation (greater than 90% chance) but may indicate Carney Triad. In the setting of GIST, Carney Triad is more likely, but SDHB, SDHC or SDHD mutation accounts for at least 25% of type 2 GIST.

**Table 1:** Syndromes associated with paraganglioma and pheochromocytoma

Syndrome	Gene	Incidence	Clinical syndrome
Von Hippel-Lindau syndrome	VHL(3p25)	1 in 36000	Retinal/CNS Hemangioblastoma Conventional clear cell renal carcinoma Phaeochromocytomas Endolymphatic sac tumours Pancreatic serous cystadenomas Pancreatic neuroendocrine tumours Epidymal/broad ligament papillary cystadenomas
Multiple endocrine neoplasia type 2	RET(10q11.2)	2.5 per 100 000	Medullary thyroid carcinoma Phaeochromocytomas Parathyroid hyperplasia
Neurofibromatosis type 1	NF1(17q11.2)	1 in 3000	Neurofibromas Café au lait spors Gliomas Lisch Nodules
Paraganglioma Syndrome type 1 (PGL1)	SDHD(11q23)	???	Pheochromocytomas/Paragangliomas Most common locations: 1. Head and neck 2. Adrenal 3. Intraabdominal extra-adrenal 4. Thorax Type 2 GIST Renal Tumors
Paraganglioma Syndrome Type 2	??SDHAF2(11q13.1)	Extremely Rare	Head and neck paragangliomas
Paraganglioma Syndrom Type 3 (PGL3)	SDHC(1q21-23)	??? (Rare)	Head and neck paragangliomas Renal tumours Type 2 GIST
Paraganglioma Syndrome Type 4 (PGL4)	SDHB(1p35-36)	???	Pheochromocytomas/Paragangliomas Increased risk of malignant behaviour Most common locations: 1. Intraabdominal extra-adrenal 2. Adrenal 3. Head and neck 4. Thorax Renal Tumours Type 2 GIST
Carney Triad	No known familial case No known mutation	Extremely rare	1.Paragagnlioma 2. ‘Type 2 Gist’ 3. Pulmonary chondroma 4??Oesophageal leiomyoma?? 5??Adrenal adenoma??

The mitochondrial complex 2 links the Krebs cycle and the electron transport chain and is illustrated below:

**Figure 1:**